# The Dandelion

**Published:** 1891-07

**Authors:** 


### THE DANDELION.

The common wild dandelion, with its bright, golden blossom, that comes to gladden our eyes in the early spring, is familiar to every child who has roamed fields and pastures. But it is not valued as it would be, if better known, for its medicinal qualities. It may be used in several ways. The salad is one of the most healthful that can be served in the early spring. It is also very palatable wilted and dressed with melted butter and vinegar. In some places it is used as greens, and esteemed highly when cooked with bacon and served with egg sauce. Of late years this modest little plant is cultivated in the hot-houses of large cities, and may be found in the markets all winter. But the wild dandelions of the field will be found much more delicate in flavor. To have early dandelions for greens and salads, people living in the country can select a place where they are grown the season before and cover with flat stones or planks, and let remain there for ten or fifteen days—this may be done very early in the spring. When uncovered, the little plants will be found tender and of a golden color.

